# The prevalence of hepatitis C and B among patients on hemodialysis and on renal transplantation waiting list in Poland has significantly decreased during the last 10 years

**DOI:** 10.1007/s11255-018-1897-7

**Published:** 2018-06-04

**Authors:** Jolanta Malyszko, Jacek Zawierucha, Wojciech Marcinkowski, Tomasz Prystacki, Teresa Dryl-Rydzynska, Jacek S. Malyszko, Joanna Matuszkiewicz-Rowinska

**Affiliations:** 1 Department of Nephrology, Dialysis and Internal Medicine, Warsaw Medical University, Banacha 1a, 02-097 Warsaw, Poland; 2 Fresenius Medical Care, Poznan, Poland; 3 1st Department of Nephrology and Transplantology with Dialysis Unit, Medical University, Białystok, Poland

Hepatitis B virus (HBV) and hepatitis C (HCV) infections are a global public health problem.

Approximately 2 billion people worldwide have evidence of past or present infection with HBV, and it is estimated that 248 million individuals are chronic carriers [i.e., positive for hepatitis B surface antigen (HBsAg)] [1]. Prevalence of HBsAg is reported to be 3.6%; however, it varies depending upon the geographic area. Roughly 600,000 die annually from HBV-related liver disease [1]. HBsAg positivity rates in dialysis patients, although significantly decreased over the several years, correlate with endemicity in the general population with 1% in US [2] through 1.3–14.6% in Asian Pacific countries [3]. On the other hand, it is estimated that 130–150 million individuals worldwide (representing 2–3% of the world population) are chronically infected with HCV and that 350,000–500,000 of these die each year from long-term complications of this infection, i.e., cirrhosis and hepatocellular carcinoma [4, 5]. The prevalence of HCV among patients with chronic kidney disease (CKD) is substantially higher than in the general population, ranging from 10 to 50%, depending on the geographical area [6] (e.g., 7–40% in developed countries [7] and 3–20% in Western European countries [8]). Recently, Goodkin and Bieber [9] described the international prevalence of HCV among hemodialysis patients awaiting transplantation. In the Dialysis Outcomes and Practice Patterns Study (DOPPS) database, this prevalence varied from 0 in China and France to 4.8% in the US and to 11% in the Gulf Cooperation Council countries.

**Fig. 1:**
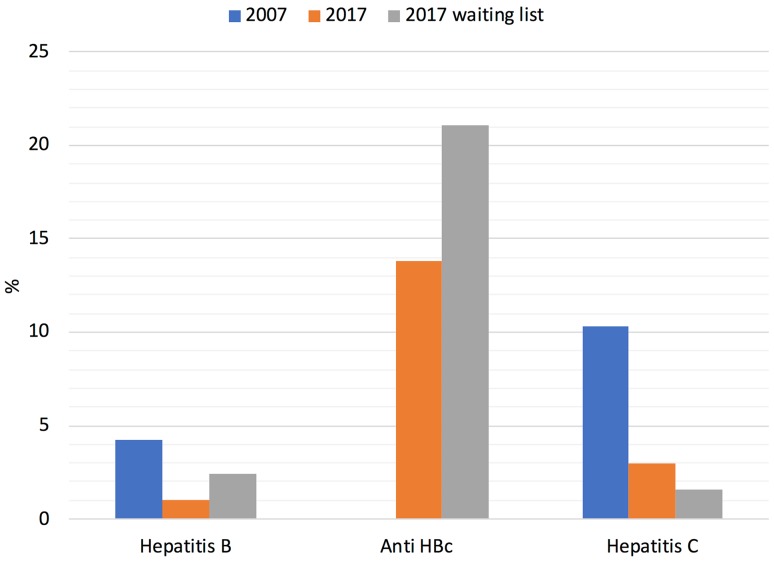
Prevalence of hepatitis B, hepatitis C, and anti-HBc antibodies (%) in hemodialyzed patients in 2007 and 2017 and on the waiting list for kidney transplantation in 2017

In 2017, we conducted a study on 300 potential kidney transplant recipients from 26 dialysis centers in Poland, representing 9.7% of all dialysis patients in these units [10]. We found that hepatitis B virus (HBV) and HCV taken together were more prevalent in patients on the inactive waiting list compared to those on the active list (3.0 vs. 1.5%, *p* < 0.05). We also looked at the prevalence of HBV and HCV, as well as that of anti-HBc antibodies, in patients from Fresenius Medical Care dialysis units (*n* = 5890, representing 1/3 of the whole hemodialysis population in Poland). We then compared the results with similar data from 2007 and with data from the transplantation waiting list in 2017 (Fig. 1). On the waiting list, HBs+/HBV−DNA+ were found in 0.5%, HBs+/HBV−DNA− in 0.5%, anti-HCV−/HCV−RNA+ in 0.2%, and anti-HBc in 21.1% of the patients. In 2007, anti-HBc was not tested. According to our results, the prevalence of HCV in Polish dialysis and waitlisted populations is comparable to that in the general population worldwide [5] and much lower than that in the US [9]. This could be due to the very strict infection control policy in our dialysis units, including the use of separate machines and rooms for HBV and HCV positive patients, as well for patients with anti-HBc antibodies. It also appears that, thanks to vaccination, the prevalence of HBV has declined. In our population, prevalence of HBV infection was similar to that of US [2], Malaysia and Japan from Asian countries [3], but much lower than in China, Thailand and Korea [3].

With modern treatment of HCV using direct-acting antiviral (DAA) drugs, sustained viral response rate may reach 100% [11]. Thus, the cure of HCV in CKD stages 4–5, dialysis, and kidney transplant patients now seems possible, and the prevalence of HCV will likely decline rapidly in the near future. Moreover, the option of DAA treatment prior to transplantation has the advantages of shortened waiting times and expansion of the organ donor pool to include HCV+ donors [12]. However, there is a risk of DAA interaction with several other drugs, including calcineurin inhibitors [13]; thus, the timing of antiviral therapy for waitlisted patients (before vs after transplantation) should be decided in collaboration with the transplant center.
